# The Potential Diagnostic Utility of SMAD4 and ACCS in the Context of Inflammation in Type 2 Diabetes Mellitus Patients

**DOI:** 10.3390/biomedicines12092015

**Published:** 2024-09-04

**Authors:** Habiba Khdair Abdalsada, Yusra Sebri Abdulsaheb, Samaneh Zolghadri, Hussein Kadhem Al-Hakeim, Agata Stanek

**Affiliations:** 1 Department of Clinical Laboratory Sciences, College of Pharmacy, Al-Muthanna University, Al-Muthanna 66001, Iraq; habiba.khdair@mu.edu.iq; 2 Clinical Pharmacy Department, College of Pharmacy, Missan University, Missan 62001, Iraq; yusrachemist@uomisan.edu.iq; 3 Department of Biology, Jahrom Branch, Islamic Azad University, Jahrom 7414785318, Iran; z.jahromi@ut.ac.ir; 4 Department of Chemistry, Faculty of Science, University of Kufa, Najaf 54001, Iraq; 5 Department and Clinic of Internal Medicine, Angiology and Physical Medicine, Faculty of Medical Sciences in Zabrze, Medical University of Silesia, Batorego 15 St, 41-902 Bytom, Poland

**Keywords:** Sma mothers against decapentaplegic homolog-4 (SMAD4), 1-aminocyclopropane-1-carboxylate synthase (ACCS), granulocyte–colony-stimulating factor (G-CFS), diabetes mellitus, insulin resistance

## Abstract

The search for new parameters for the prediction of type 2 diabetes mellitus (T2DM) or its harmful consequences remains an important field of study. Depending on the low-grade inflammatory nature of diabetes, we investigated three proteins in T2DM patients: 1-aminocyclopropane-1-carboxylate synthase (ACCS), granulocyte–colony-stimulating factor (G-CSF), and Sma Mothers Against Decapentaplegic homolog-4 (SMAD4). In brief, sixty T2DM and thirty healthy controls had their serum levels of ACCS, G-CSF, SMAD4, and insulin tested using the ELISA method. The insulin resistance (IR) parameter (HOMA2IR), beta-cell function percentage (HOMA2%B), and insulin sensitivity (HOMA2%S) were all determined by the Homeostasis Model Assessment-2 (HOMA2) calculator. The predictability of these protein levels was investigated by neural network (NN) analysis and was associated with measures of IR. Based on the results, ACCS, G-CSF, and SMAD4 increased significantly in the T2DM group compared with the controls. Their levels depend on IR status and inflammation. The multivariate GLM indicated the independence of the levels of these proteins on the covariates or drugs taken. The receiver operating characteristic area under the curve (AUC) for the prediction of T2DM using NN analysis is 0.902, with a sensitivity of 71.4% and a specificity of 93.8%. The network predicts T2DM well with predicted pseudoprobabilities over 0.5. The model’s predictive capability (normalized importance) revealed that ACCS is the best model (100%) for the prediction of T2DM, followed by G-CSF (75.5%) and SMAD4 (69.6%). It can be concluded that ACCS, G-CSF, and SMAD4 are important proteins in T2DM prediction, and their increase is associated with the presence of inflammation.

## 1. Introduction

According to the International Diabetes Federation’s estimation in 2021, the global prevalence of type 2 diabetes mellitus (T2DM) was 10.5%, with a projected increase of 12.2% by 2045. This projection indicates that over 783 million adults will be affected. Diabetes has a substantial impact on both individual and global health economies, as evidenced by estimated costs of USD 966 billion [1]. Individuals with T2DM are highly susceptible to morbidity and mortality, primarily due to cardiovascular disease. Attaining rigorous glycemic control is crucial in mitigating the probability of complications and diabetes disease progression [2]. T2DM is a medical condition characterized by insufficient insulin production and release by the β-cells in the pancreatic islets, as well as decreased insulin sensitivity, commonly known as insulin resistance (IR). The gradual increase in fasting and postprandial blood glucose levels is a characteristic feature of the development of this disease. It is noteworthy that a reduction in insulin sensitivity is one of the earliest pathogenic events that occur several decades prior to the onset of T2DM [3]. IR is a prevalent pathology in both obesity and T2DM. It is characterized by a cellular state in which insulin fails to elicit a response, ultimately leading to the onset of hyperglycemia [4]. The occurrence of IR results in an elevation of glucose concentration that falls within the range between normal levels and the established thresholds for T2DM. This particular metabolic state is commonly referred to as prediabetes. Although not classified as a clinical entity, it serves as a risk factor for progressing to diabetic pathology [5]. More than 90% of all diabetes cases are attributable to T2DM, which is characterized by IR and reduced cell function [6].

Many parameters were previously studied for diagnosis, prognosis, and follow-up. However, there are essential compounds that are not well-researched. The Sma Mothers Against Decapentaplegia homolog-4 (SMAD4) is a key transcriptional mediator of transforming growth factor-β (TGFβ) [7]. Since SMAD4 controls the canonical route of TGFβ, it is especially vulnerable to the effects of intermittent hypoxia and disruptions in its circadian rhythm [8]. Patients with hypertension, T2DM, and cardiovascular disease have been shown to have high plasma levels of TGFβ, indicating a probable involvement in the development of these diseases [9,10]. Therefore, for example, the pleiotropic effects of TGF signaling on energy balance and metabolism are pertinent for the genesis and development of T2DM [11]. When Smad4 is activated, it binds with other Smad proteins, moves into the nucleus, and controls the production of genes that are involved in cellular responses to oxidative stress [12]. It also plays an important role in the inflammation process [13], which causes many of the complications and symptoms of diabetes. Smad4 plays a vital role in the growth and proper functioning of pancreatic beta cells. It aids in the regulation of genes that are involved in the release of insulin and the survival of beta cells [14].

The granulocyte–colony-stimulating factor (G-CSF) is an additional biomarker, synthesized by numerous cell types, that warrants investigation in patients with T2DM. The primary role of G-CSF is to facilitate neutrophil differentiation and proliferation within the bone marrow and modulate the release of these cells into the bloodstream. Furthermore, G-CSF has been observed to have the potential to impact the oxidative process and phagocytosis [15]. Research found that persons with type 2 diabetes (T2DM) had significantly elevated blood G-CSF levels compared with the healthy controls. These levels were strongly correlated with several inflammatory indicators linked to problems associated with diabetes [16]. Another research presented data indicating that diabetic individuals had elevated levels of G-CSF, revealing its potential as a biomarker associated with inflammation and perhaps the severity of diabetes complications [17]. Previous studies have shown that elevated levels of G-CSF may suggest a continuous immunological response in patients with diabetes, indicating the presence of chronic low-grade inflammation often seen in diabetics [18].

The 1-Aminocyclopropane-1-carboxylate synthase enzyme (ACCS), also known as S-adenosyl-L-methionine methylthioadenosine lyase, catalyzes the conversion of SAM into 1-aminocyclopropane-1-carboxylate (ACC) [19]. The ACCS regulates the biosynthesis of ethylene [20], in addition to many other pathological processes, including cancer, infection, and inflammation [21,22,23]. The intestinal microbiota is believed to increase levels of ethylene, the precursor of ethylene oxide, through metabolic processes and lipid peroxidation [24]. However, the role of ACCS in diabetes has not yet been studied.

In the present study, ACCS, GCSF, and SMAD4 levels in T2DM were correlated with IR status, and their ability to diagnose diabetes mellitus state using neural network (NN) analysis was studied.

## 2. Materials and Methods

### 2.1. Subjects

The present investigation enlisted a cohort of 60 individuals diagnosed with T2DM and 30 individuals who were considered healthy controls, with age and sex being matched to the T2DM patients. This study recruited participants from Sadr Teaching Hospital, Najaf Governorate, Iraq, during the period from December 2022 to the end of January 2023. The diagnostic criteria for T2DM patients followed the guidelines set forth by the World Health Organization [25,26], which stipulates that patients must exhibit fasting plasma glucose levels of ≥7.0 mM and the level of glycated hemoglobin (HbA1c) exceeding 6.5%.

The exclusion criteria employed for patients in this study were as follows: patients who exhibited major overt diabetic complications, including heart disease, liver disease, and renal diseases, as well as patients whose albumin/creatinine ratio exceeded 30 mg/g [27]. Serum CRP concentrations of all participants were found to be below 6 mg/dL, indicating the absence of overt inflammation [28]. The participants under consideration engage in physical exercise by visiting a fitness center at least twice a week and dedicating a minimum of one hour to training during each visit. All participants gave their informed written permission to take part in this study before they were enrolled. The IRB of the University of Kufa gave its permission to carry out the research (IRB reference number 811B/2022). As required by the Declaration of Helsinki, the IRB follows the International Guidelines for the Protection of Human Subjects of Research.

### 2.2. Methods

Blood was collected at 8:00 a.m. after an overnight fasting period. Venous blood samples were collected from both patients and controls, with a volume of five milliliters per sample. After complete clotting, the blood sample was centrifuged at a rate of 1500× *g* for 10 min. The resulting serum was then separated and stored at a temperature of −80 degrees Celsius until further analysis. Serum levels of ACCS, SMAD4, and G-CSF were quantified by ELISA reader (Model, Biotek ELx 800, Hudson, NY, USA) using commercial ELISA sandwich kits obtained from Nanjing Pars Biochem Co., Ltd. (Nanjing, China). The procedures were executed precisely as per the manufacturer’s guidelines, without any alterations. The limit of detection for the other kits was 1 ng/mL. The precision within an assay, as indicated by the intra-assay coefficient of variation (CV), was found to be less than 10.0%.

Fasting blood glucose was measured by spectrophotometer (Model CGOLDENWALL 721, Guangzhou, China) using a kit supplied by Agappe^®^ Diagnostics Ltd. (Cham, Switzerland). The kit used to detect serum CRP levels was supplied by Spinreact^®^ (Girona, Spain). The Homeostasis Model Assessment 2 (HOMA2) calculator© (Version 2.2.3), developed by University of Oxford (UK), downloaded freely at https://www.dtu.ox.ac.uk/homacalculator/download.php (14 February 2022), was employed to derive measures of β-cell function (HOMA2%B), insulin sensitivity (HOMA2%S), and insulin resistance (HOMA2-IR) based on the individual’s fasting serum insulin and glucose concentrations. The HOMA2 calculator uses a more complex algorithm that accounts for variations in fasting insulin and glucose levels. HOMA2 provides estimates of both IR and HOMA2%B, offering a more comprehensive view of metabolic health compared with the simple HOMA-IR equation, which only focuses on insulin resistance [29]. Studies have shown that HOMA2 calculator results correlate better with gold-standard measures of insulin sensitivity, such as the euglycemic clamp technique [30].

The exclusion criteria for patients consisted of serum fasting blood glucose levels exceeding 25 mM and fasting insulin levels surpassing 400 pM, as determined by the HOMA calculator software (Version 2.2.3). Furthermore, patients with overt diabetic complications and heart, liver, and kidney diseases were excluded. We excluded patients receiving metformin due to its potential impact on IR measurement [30] and insulin sensitivity [31], as well as patients with an albumin/creatinine ratio greater than 30 mg/g [27]. All participants had serum CRP concentrations below 6 mg/dL, indicating the absence of overt inflammation among the subjects. The participants under consideration engage in physical exercise by visiting a fitness center at a minimum frequency of twice per week and allocating a minimum of one hour per visit for training purposes.

### 2.3. Statistical Analysis

The Kolmogorov–Smirnov test was utilized to analyze the distribution types of the variables. The mean ± standard deviation was used to express the results for variables that followed a normal distribution. Analysis of variance (ANOVA) was used to quantify differences between continuous variables, while the analysis of contingency tables (χ^2^-test) was used to assess correlations between nominal variables. Results were reported as medians with an interquartile range of 25% to 75% when the variables did not follow a normal distribution. The Mann–Whitney test was used to make comparisons between the patient and control cohorts, as well as within subgroups, with respect to the measured parameters. The Spearman correlation coefficients (ρ, rho) were calculated in order to assess the correlation between parameters. This study used multivariate general direct model (GLM) analysis to examine the correlation between biomarkers and diagnosis (controls versus T2DM), while also accounting for background variables such as age, BMI, nicotine dependence, sex, and education. This study conducted tests to examine the associations between diagnosis and each biomarker through between-subject effects. The effect sizes were evaluated using partial eta (η^2^) values. The sample size computation was conducted using the G*Power 3.1.9.7 software. Based on the utilization of an effect size of 0.3, a *p*-value of 0.05, a power of 0.8, and the incorporation of two groups in the analysis of covariance, it is recommended that the overall sample size be 90. Consequently, a total of 90 participants were recruited, comprising 30 individuals in the control group and 60 patients in the experimental group.

To ensure normal distribution for all statistical analyses, the variables SMAD4, ACCS, and G-CSF underwent the Ln transformation. The statistical tests used were two-tailed, and the threshold for statistical significance was set at a *p*-value of 0.05. Statistical analyses were performed using IBM SPSS Windows version 25 in 2017.

This study used Multilayer Perceptron NN models to evaluate the intricate relationships between the diagnosis of T2DM and the control group as output variables, and the measured biomarkers as input variables. The network was trained using automated feedforward architecture models, which involved the utilization of two hidden layers with a maximum of four nodes. Minibatch training with gradient descent was used and the training process was carried out over 30 epochs. Ultimately, error, relative error, and importance were calculated for all input variables. The statistical tests employed were two-tailed and the level of significance chosen was 0.05.

## 3. Results

### 3.1. Sociodemographic and Clinical Characteristics

Table 1 shows the clinical and biochemical data of healthy controls and T2DM patients. Age, BMI, sex, marital status, residency, TUD, and exercise frequency are not significantly different between patients and control groups. Patients treated with Sitagliptin (100 mg) or Amaryl (4 mg) were 18 out of 60, while 24 patients were treated with Daonil (5 mg). The patient’s group has a significantly higher (*p* < 0.001) family history of T2DM than the control group. As expected, there is significant hyperglycemia in T2DM patients. The duration of disease in the patient’s group was 12.411 ± 5.363 years. The results show a significant decrease (*p* < 0.001) in HOMA2%B and HOMA2IR, in addition to a significant increase in HOMA2%S in T2DM patients compared with the control group. Serum levels of SMAD4, ACCS, and G-CSF are significantly higher (*p* < 0.001) than in the control group.

### 3.2. Results of Multivariate GLM Analysis

Table 2 shows the results of a multivariate GLM analysis with measured biomarkers as dependent variables, while diagnosis and covariates (age, BMI, exercise, sex, and TUD) were the explanatory variables. There was a highly significant effect of diagnosis (F = 12.959, df = 9/75, *p* < 0.001) with an effect size (Partial η^2^) of 0.637 on the level of biomarkers. All covariates had no significant effect on biomarkers.

Tests for effects between subjects showed that diagnosis has significant effects on serum biomarker levels. The six main biomarkers that significantly (*p* < 0.001) affected by the diagnosis were insulin/glucose ratio (F = 71.983, Partial η^2^ = 0.464), HOMA2%B (F = 71.811, Partial η^2^ = 0.487), ACCS (F = 40.722, Partial η^2^ = 0.329), insulin (F = 36.126, Partial η^2^ = 0.303), SMAD4 (F = 35.426, Partial η^2^ = 0.299), and GCSF (F = 30.418, Partial η^2^ = 0.268), respectively.

### 3.3. Correlation Matrix

The correlation matrix showing the partial correlations of IR parameters indices, SMAD4, ACCS, and G-CSF with other measured parameters adjusted for age, sex, BMI, smoking, and exercise are presented in Table 3. ACCS is correlated with glucose (r = 0.273, *p* < 0.01), G-CSF (r = 0.370, *p* < 0.01), and SMAD4 (r = 0.430, *p* < 0.001), while ACCS is inversely and significantly correlated with insulin (r = −0.221, *p* < 0.05), insulin/glucose ratio (r = −0.282, *p* < 0.05), and HOMA2%B (r = −0.292, *p* < 0.01). G-CSF is significantly correlated with glucose (r = 0.344, *p* < 0.001), HOMA2%S (r = 0.214, *p* < 0.05), ACCS (r = 0.370, *p* < 0.01), and SMAD4 (r = 0.745, *p* < 0.001).

Serum G-CSF is significantly and inversely correlated with insulin (r = −0.274, *p* < 0.01), In/G (r = −0.336, *p* < 0.001), HOMA2%B (r = −0.336, *p* < 0.01), and HOMA2IR (r = −0.214, *p* < 0.05). A significant correlation between SMAD4 with glucose (r = 0.341, *p* < 0.001) and ACCS (r = 0.430, *p* < 0.001). Serum SMAD4 is significantly and inversely correlated with HOMA2%B (r = −0.346, *p* < 0.001), insulin (r = −0.215, *p* < 0.05), and In/G (r = −0.306, *p* < 0.01).

### 3.4. Neural Network Study

The results of the neural network information of the model in patients to predict patients with T2DM versus controls are presented in Table 4. NN analysis employed a feedforward architecture, which involves the absence of feedback loops, as the connections within the network flow exclusively from the input layer to the output layer. The present analysis involves the utilization of an input layer that comprises the predictors. The concealed stratum comprises latent nodes, also known as units, that are not directly observable. The value of the hidden unit is computed using a function that takes into account both the kind of network and the user’s preferences. The final layer in the neural network architecture is the output layer, which is responsible for generating the responses. The two indicator variables serve to illustrate the dichotomous nature of the underlying variable. Each unit’s output is calculated using a formula based on the hidden units that go into making it up. The precise structure of the function depends on both the type of network and the specifications that can be manipulated. There are three units (measured parameters) in the input layer (layer containing factors for the prediction of the T2DM). This model was trained using two hidden layers, layer 1, consisting of three units, and layer 2, consisting of two units, using the hyperbolic tangent and identity as activation functions, respectively.

**Figure 1:**
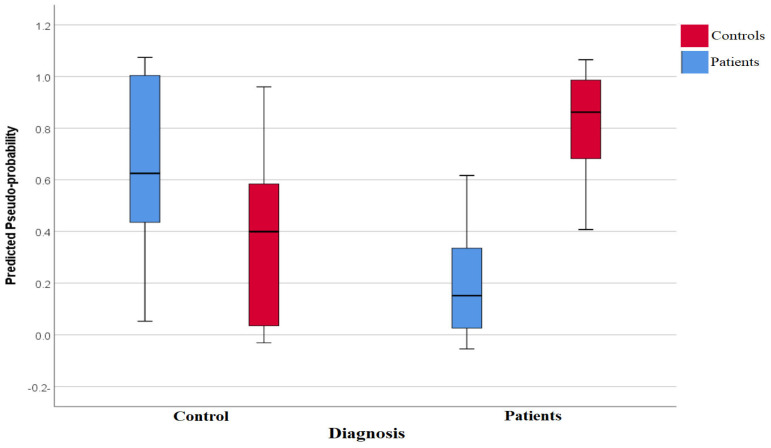
Box plot for predicted pseudoprobabilities for neural network analysis to differentiate diabetic patients from controls.

**Figure 2:**
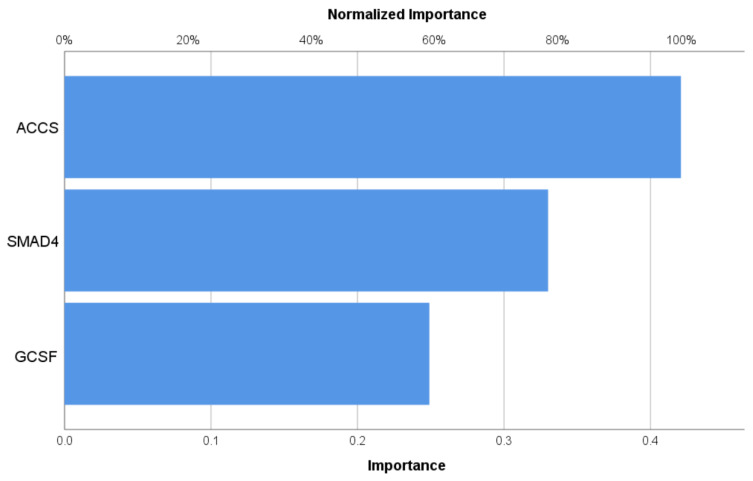
The relative importance of measured biomarkers in predicting type 2 diabetes mellitus. ACCS: 1-aminocyclopropane-1-carboxylate synthase, G-CSF: granulocyte–colony-stimulating factor, SMAD4: Sma Mothers Against Decapentaplegic homolog-4.

Pseudoprobabilities predicted from both the training and holdout data are shown in a box plot in Figure 1. The categories of responses that were actually received are shown along the x-axis, while the categories that were anticipated are labeled in the legend. Correct predictions are shown in the part of the box plot where the y-axis is greater than the 0.5 threshold. For this analysis, the individual pseudoprobabilities of having T2DM were estimated from the classifier output using sigmoid fits. The area under the curve (AUC) of the receiver operating characteristic was 0.902, with a sensitivity of 71.4% and a specificity of 93.8%, in each of the three sets of data. It is shown in Figure 2 how significant each of the model’s input variables is in terms of the model’s predictive ability. In terms of predictive capability (normalized importance), ACCS is the best model (100%) for the prediction of T2DM, followed by G-CSF (75.5%), and SMAD4 (69.6%).

## 4. Discussion

The primary discovery in the current investigation is the elevation of serum SMAD4, ACCS, and G-CSF concentrations in individuals with T2DM in contrast to the control cohort, as demonstrated in Table 1. The observed outcomes can be elucidated via various mechanisms. Hyperglycemia has been observed to disrupt the normal physiological processes of various bodily systems, including the liver, pancreatic beta cells, circulatory system, skeletal muscles, and gastrointestinal tract. This disruption can lead to systemic IR, immune cell dysfunction, heightened intestinal permeability, and increased susceptibility to infections and inflammation in individuals with T2DM [32]. Both patients and the control group are within the normal values. However, it is expected that T2DM patients have a lower insulin level than control due to the beta cell dysfunction. Before being T2DM patients, they usually have an insulin resistance state expressed as hyperinsulinemia and insulin resistance scale. After years of insulin resistance and hyperinsulinemia, the beta-cells are malfunctioned and secrete lower insulin amounts.

The investigated molecules SMAD4 [13], ACCS [33], and GCSF [34] are known as inflammation-related molecules. Inflammation is an important part of diabetes pathophysiology and complications [35,36]. Therefore, inflammation in diabetes is the plausible and acceptable cause of increased levels of the proteins ACCS, GCSF, and SMAD4. Serum CRP concentrations, measured by the latex agglutination tests, of all participants were found to be negative below 6 mg/dL, indicating the absence of overt inflammation. Therefore, there is no need to add the negative results. We used the CRP test to exclude any patients with overt inflammation (positive CRP). Therefore, T2DM patients in the present study may have low-grade inflammation.

Diabetes and IR are associated with the presence of oxidative stress, a pathological process [37] that increases the likelihood of complications associated with T2DM, such as nephropathy, neuropathy, retinopathy, and accelerated coronary artery disease [38]. Studies have indicated that elevated glucose levels can induce damage to β-cells through the activation of reactive oxygen species (ROS), ultimately resulting in impaired insulin release and IR [39]. The present study suggests that the increase in the ACCS, GCSF, and SMAD4 proteins observed in T2DM patients may be attributed to the inflammation caused by ROS.

The possible effects of the covariates (age, BMI, exercise, sex, and TUD) on the levels of the measured parameters were investigated to attribute the cause of the increase to the presence of T2DM. The results of multivariate GLM analysis are displayed in Table 2 and showed a highly significant effect of the disease with a high effect size on the levels of ACCS, GCSF, and SMAD4 biomarkers. All the covariates had no significant effects on the biomarkers and the cause for the increase in the levels of these e proteins is due to the presence of diabetes in the subjects. Tests for effects between subjects showed that diagnosis has the highest significant effects on the levels of serum Insulin/Glucose ratio, HOMA2%B, ACCS, insulin, SMAD4, and GCSF after adjusting for all the covariates.

The main general conclusions from the partial correlation results in Table 3 are the correlation of the levels of the investigated molecules (ACCS, SMAD4, and GCSF) with the biomarkers of hyperglycemia, IR, and inflammation parameters. These results indicate the role of these molecules in many areas of diabetes pathophysiology. Furthermore, SMAD4 exhibits a diverse regulatory impact on metabolic homeostasis, stimulating fibrosis, fibroblast, and matrix modification, as well as inflammation, as stated in the reference [13]. Elevated levels of SMAD4 in people with diabetes may be attributed to the correlation between hypoxemia and diabetic acidosis, which has previously been shown to increase the expression of SMAD4 [8]. The significant association between SMAD4 mRNA expression and hypoxia-inducible factor 1-alpha (HIF1α) as well as the mechanistic target of rapamycin (mTOR) mRNA expression, which plays a crucial role in hypoxia, can account for this phenomenon [8]. Dyslipidemia, which is often comorbid with diabetes, has been identified as a contributing factor to elevated levels of plasma SMAD4 [8]. Prior research has substantiated that the expression of TGFβ is induced by both HIF1α and circadian genes [40]. Also, persistent increases in insulin levels might result in abnormal glucose metabolism and lipid balance, causing a pro-inflammatory state. This leads to the production of proinflammatory cytokines from adipose tissue and overactive immune cells, which interfere with insulin signaling pathways. Inflammation disrupts insulin signaling cascades by activating kinase molecules, causing insulin resistance [41]. The release of additional inflammatory mediators and increased oxidative stress worsen insulin resistance. This condition is worsened by the initiation of immunological responses as a result of inflammation in tissues, namely adipose tissue [42]. Additionally, individuals with T2DM showed significant levels of TGF in their plasma when they participated in a prospective trial [10]. The SMAD pathway has also been shown to have a strong connection to the development of diabetes [43].

Table 4 presents the outcomes of the neural network analysis of a model designed to predict T2DM based on serum levels of G-CSF, ACCS, and SMAD4. As the network’s connections propagate forward from the input layer to the output layer, the NN study used a feedforward design, which is distinguished by the lack of feedback loops. The historical default variable is represented by two indicator variables, since it is a binary category variable. Each unit’s output is calculated using a formula based on the hidden units that go into making it up. The particular form of the function depends on the kind of network and the tunable parameters. The input layer, which comprises factors to predict the risk of T2DM consists of three units that serve as measured parameters.

The predicted pseudo-probabilities correspond to the degree to which the state of a given individual appears to match the T2DM pattern as seen in Figure 1. The network’s performance in predicting instances belonging to the fixed time category using the 0.5 cutoffs is clearly shown in the figure. Figure 2 shows the relative importance of the measured parameters to predict T2DM in a subject. The ACCS showed the highest predictability value. This result is novel and needs to be explained in detail.

Previously reported findings indicate that elevated levels of G-CSF in patients with T2DM are the result of an inflammatory response [44]. The role of G-CSF in the development of free fatty acid (FFA) induced IR has been suggested to be significant, as evidenced by the induction of IR in human adipocytes and myocytes after treatment with G-CSF [45]. According to a previous study, elevated glucose and free fatty acids elicited a significant increase in the release of IL-6, IL-8, chemokines, and G-CSF by human beta-islets [46]. The administration of G-CSF has been found to ameliorate neutropenia manifestations through its ability to promote neutrophil progenitor cell proliferation and increase the activity of fully differentiated neutrophils [47]. The administration of G-CSF has been shown to reduce the duration and severity of neutropenia, as well as the occurrence of severe and febrile neutropenias, and the mortality associated with infection in clinical contexts [48]. IR is characterized by a reduced biological response to a specific concentration of insulin, which is lower than the normal response [49].

ACCS is a pyridoxal phosphate-containing enzyme, and its activity is regulated at the transcriptional and posttranscriptional levels [21]. However, the serum level of this protein has not previously been studied. The increase in this protein in our patients may explain the presence of measurable amounts of ethylene in different consequences of diabetes.

The detection of ethylene preceded the elevation of inflammatory cytokines and stress-related hormones in humans. The aforementioned findings have brought to attention the importance of ethylene emission as a fundamental and premature element of in vivo lipid peroxidation, which has crucial clinical implications as a breath-based biomarker for bacterial infection [22]. Several scholars have proposed that ethylene may be generated in vivo as a result of oxidative harm [23,50,51]. The release of ethylene was observed to be a component of the larger inflammatory and stress response, as evidenced by its significant correlation with the release of epinephrine and IL-10 [22]. According to research, ethylene has been proposed to be discharged by humans during systemic inflammation through blood leukocytes, which is achieved through the oxidation of unsaturated fatty acids or other related molecules [22]. Moreover, there exists evidence that ethylene release prior to inflammatory cytokines presents a promising prospect for its participation in the initiation of the cytokine response [52]. Previous research has established a correlation between ethylene and oxidative stress in mammals [23,50]. The presence of oxidative stress among people with T2DM triggers the production of inflammatory mediators. This, in turn, leads to an increase in ROS generation among diabetic patients.

This interaction between, oxidative stress, inflammation, and ethylene production may act as a promotor for the increase in the ACCS concentration in T2DM patients. However, these results would be strictly confirmed if we could measure the ethylene concentration in diabetic patients. The main limitation of this study is the relatively moderate sample size. Also, it would be better to measure the glycated hemoglobin as another biomarker for the level of the measured parameters.

## 5. Conclusions

The increased serum levels of ACCS, G-CSF, and SMAD4 in the T2DM group could be used for the prediction of T2DM disease by using NN analysis. These protein levels depend on the IR status and inflammation, regardless of the covariates of the drugs taken. NN analysis produces good sensitivity and specificity for the prediction of T2DM. The model’s predictive capability (normalized importance) revealed that ACCS is the best model (100%) for the prediction of T2DM, followed by G-CSF (75.5%) and SMAD4 (69.6%).

## Figures and Tables

**Table 1:** Demographic, clinical, and biochemical data in type 2 diabetes mellitus (T2DM) patients and healthy controls.

Variables		Control n = 30	T2DM n = 60	df	F/χ^2^	*p*
Age	years	44.67 ± 9.279	46.62 ± 8.279	1/88	1.023	0.315
Sex	(Female/Male)	19/11	37/23	1	0.024	0.878
BMI	(kg/m^2^)	26.778 ± 4.819	26.837 ± 3.052	1/88	0.005	0.944
Single/Married		2/28	4/56	1	0	1
Residency Rural/Urban		3/27	6/54	1	0	1
Family History	(No/Yes)	29/1	35/25	1	14.306	<0.001
Exercise	(No/Yes)	8/22	11/49	1	1.092	0.311
TUD	(No/Yes)	19/11	34/26	1	0.367	0.545
Duration of DM	Years	-	12.411 ± 5.363	-	-	-
Glucose	mM	5.532 ± 0.686	8.182 ± 2.742	1/88	27.038	<0.001
Insulin	pM	60.228 ± 10.731	42.592 ± 13.171	1/88	40.329	<0.001
(Insulin/Glucose) ×10^−9^		11.018 ± 2.324	5.884 ± 2.774	1/88	75.975	<0.001
HOMA2%B		83.250 (71.825–102.925)	37.650 (21.650–57.625)	1/88	75.008	<0.001
HOMA2%S		86.800 (76.025–102.000)	114.850 (91.825–135.850)	1/88	14.652	<0.001
HOMA2IR		1.151 ± 0.208	0.884 ± 0.252	1/88	25.136	<0.001
SMAD4	ng/mL	63.335 (34.757–106.058)	177.793 (84.827–284.376)	1/88	37.916	<0.001
ACCS	pg/mL	4.0188 (3.149–5.965)	6.061 (5.680–7.303)	1/88	39.313	<0.001
G-CSF	pg/mL	67.059 (34.344–110.080)	143.992 (89.753–210.583)	1/88	30.207	<0.001
Sitagliptin 100 mg	(No/Yes)	30/0	42/18	1	11.250	0.001
Amaryl 4 mg	(No/Yes)	30/0	42/18	1	11.250	0.001
Daonil 5 mg	(No/Yes)	30/0	36/24	1	16.364	<0.001

ACCS: 1-aminocyclopropane-1-carboxylate synthase, BMI: body mass index, G-CSF: granulocyte-colony stimulating factor, HOMA2IR: Homeostasis Model Assessment 2 insulin resistance score, HOMA2%B, Beta cell function percentage, HOMA2%S: insulin resistance percentage, SMAD4: Sma Mothers Against Decapentaplegic homolog-4, TUD: tobacco use disorder.

**Table 2:** Results of multivariate GLM analysis examining the associations between the measured biomarkers and the diagnosis (healthy control/T2DM).

Test	Dependent Variables	Explanatory Variables	F	df	*p*	Partial η^2^
Multivariate	All measured biomarkers	Diagnosis	12.959	9/75	<0.001	0.637
		Age	0.721	9/75	0.702	0.089
		Sex	1.258	9/75	0.288	0.156
		TUD	0.575	9/75	0.829	0.072
		Exercise	0.485	9/75	0.894	0.062
		BMI	1.023	9/75	0.432	0.122
Between-subject effects	Diagnosis	Insulin/Glucose	71.983	1	<0.001	0.464
		HOMA2%B	71.811	1	<0.001	0.487
		LnACCS	40.722	1	<0.001	0.329
		Insulin	36.129	1	<0.001	0.303
		LnSMAD4	35.426	1	<0.001	0.299
		LnGCSF	30.418	1	<0.001	0.268
		Glucose	27.320	1	<0.001	0.248
		HOMA2IR	21.768	1	<0.001	0.208
		HOMA2%S	12.629	1	0.001	0.132

ACCS: 1-aminocyclopropane-1-carboxylate synthase, BMI: body mass index, G-CSF: granulocyte-colony stimulating factor, HOMA2IR: Homeostasis Model Assessment 2 insulin resistance score, HOMA2%B, Beta cell function percentage, HOMA2%S: insulin resistance percentage, Ln: natural logarithm, SMAD4: Sma Mothers Against Decapentaplegic homolog-4, TUD: tobacco use disorder.

**Table 3:** Correlation matrix showing the partial correlations adjusted for age, sex, BMI, smoking, and exercise.

	ACCS	G-CSF	SMAD4
Glucose	0.273 **	0.344 **	0.341 **
Insulin pM	−0.221 *	−0.274 **	−0.215 *
In/G	−0.282 **	−0.336 **	−0.306 **
HOMA2%B	−0.292 **	−0.356 **	−0.346 **
HOMA2%S	0.176	0.214 *	0.175
HOMA2IR	−0.176	−0.214 *	−0.175
ACCS	1.000	0.370 **	0.430 **
G-CSF	0.370 **	1.000	0.745 **
SMAD4	0.430 **	0.745 **	1.000

*: *p* < 0.05, **: *p* < 0.01, ACCS: 1-aminocyclopropane-1-carboxylate synthase, G-CSF: granulocyte-colony stimulating factor, HOMA2IR: Homeostasis Model Assessment 2 insulin resistance score, HOMA2%B, Beta cell function percentage, HOMA2%S: insulin resistance percentage, SMAD4: Sma Mothers Against Decapentaplegic homolog-4.

**Table 4:** Results of neural networks with type 2 diabetes mellitus and healthy controls as a reference group.

	Models	T2DM vs. Control
Input Layer	Number of units	3
	Rescaling method	Normalized
Hidden layers	Number of hidden layers	2
	Number of units in hidden layer 1	1
	Number of units in hidden layer 2	2
	Activation Function	Hyperbolic tangent
Output layer	Dependent variables	T2DM vs. Control
	Number of units	2
	Activation function	Identity
	Error function	Sum of squares
Training	Sum of squares error term	7.36
	% Incorrect or relative error	22.0%
	Prediction (sensitivity, specificity)	50%, 91.0%
Testing	Sum of squares error	2.15
	Percent Incorrect Predictions	13.0%
	Prediction (sensitivity, specificity)	71.4%, 93.8%
	AUC ROC	0.902
Holdout	Percent Incorrect Predictions	11.8%
	Prediction (sensitivity, specificity)	85.7%, 90.0%

AUC ROC: area under the curve of receiver operating curve.

## Data Availability

The data presented in this study are available on request from the corresponding authors due to sensitive information (patients’ clinical data).
